# DGAT1 Mutation Associated With Congenital Diarrhea in a Pediatric Patient: A Case Report

**DOI:** 10.7759/cureus.65119

**Published:** 2024-07-22

**Authors:** Asim Mehmood, Rida Inam, Nimra Rabbani, Yasser Masood

**Affiliations:** 1 Internal Medicine, Shifa College of Medicine, Islamabad, PAK; 2 Pediatrics, Shifa International Hospital, Islamabad, PAK

**Keywords:** malnourishment, genetic testing, enteropathy, case report, congenital diarrhea

## Abstract

Chronic diarrhea in infants can stem from various etiologies, including congenital disorders affecting intestinal function. Here, we present a case of a one-year-old boy with persistent watery diarrhea, vomiting, and failure to thrive, ultimately diagnosed with DGAT1 deficiency through genetic testing. Despite initial investigations ruling out common causes like celiac disease, genetic analysis confirmed DGAT1 enteropathy. Management included intensive nutritional support and close monitoring, resulting in clinical improvement. This case underscores the importance of early genetic testing and tailored management in congenital enteropathies to prevent severe complications and improve patient outcomes.

## Introduction

There are several different factors that might contribute to chronic diarrhea, such as viral infections, post-infectious, and allergic reactions. Chronic diarrhea can also have anatomical reasons, such as volvulus causing short-bowel syndrome, gastroschisis, and necrotizing enterocolitis. Congenital diarrhea and enteropathies, a subset of chronic diarrheas, are characterized by severe clinical symptoms that typically begin with diarrhea after birth or in the early stages of infancy [1]. Only a small percentage of cases with congenital diarrhea and diverse enteropathies receive a conclusive diagnosis, frequently requiring a protracted examination process. To attain the proper development and nutritional targets, these cases frequently necessitate large and comprehensive treatments, including tailored meal regimens and parenteral nutritional support [2]. The enzyme known as diacylglycerol acyltransferases (DGATs), which catalyze the last stage of triglyceride production, is encoded by the gene DGAT. DGAT1 is widely expressed, with the human gut expressing it the highest [3]. Patients with DGAT1 deficiency experience diarrhea, vomiting, and protein-losing enteropathy, highlighting how crucial this mechanism is to maintaining intestinal homeostasis [4]. Rather than being merely a point of entry, the intestine may consequently play a significant regulatory role in systemic lipid metabolism.

## Case presentation

A one-year-old boy presented to the pediatrics outpatient department with the complaint of recurrent episodes of watery diarrhea since the 40th day of life. The diarrhea was foul-smelling and yellow in color, and the child had eight to 10 episodes per day. It was associated with vomiting, reduced oral intake, and failure to thrive. There was no evidence of pus or blood in stools. It was not associated with fever. He is fully immunized according to the Extended Program of Immunization (EPI) until his age. He was breastfed till four months of age after which he was shifted to formula milk due to recurrent diarrhea. Different types of formula milk were tried, but none of them improved his condition. His past medical history is significant for multiple hospital visits due to diarrhea, as well as an episode of pneumonia for which he was hospitalized and was given appropriate treatment leading to discharge from the hospital. His surgical history is insignificant. Moreover, he is allergic to vancomycin.

Upon examination, a pale-looking, visibly emaciated child was lying on the bed, with stable vital signs. He had the pallor of the mucosa and tenting of the skin, consistent with anemia and dehydration. His weight and height were both under the fifth percentile. Serum electrolytes, ferritin levels, phosphorus, and thyroid-stimulating hormone (TSH) levels were within the normal range. Renal function tests were within normal limits; however, liver function tests (LFTs), complete blood count (CBC), alkaline phosphatase, blood urea nitrogen, and several other tests were abnormal (Table 1). Stool and urine routine exams did not reveal any positive findings. Furthermore, immunoglobulin levels (IgA, IgG, and IgM) were also within the normal ranges.

**Table 1:** Blood tests with positive results.

Test	Result	Normal Range
Hemoglobin (g/dL)	9.5	10.5-14
Hematocrit (%)	28.7	32-42
Mean Corpuscular Volume (fL)	69.33	72-88
Mean Corpuscular Hemoglobin Concentration (pg)	22.2	32-36
Platelet Count (/microL)	426,000	150,000-400,000
Monocytes (%)	10	5-7
Eosinophils (%)	4	1-3
Cross-Reactive Protein (mg/L)	30.50	Up to 5
Alkaline Phosphatase (U/L)	459	Up to 335
Alkaline Phosphatase Fractionation (U/L)	503	40-130
Blood Urea Nitrogen (mg/dL)	3	5-18
25-Hydroxyvitamin D (ng/mL)	<3	<20
Serum Calcium (mg/dL)	8.5	9-11
Serum Albumin (g/dL)	3.0	3.8-5.4
Free T4 (ng/dL)	0.59	0.7-1.70

Various differential diagnoses were considered, including celiac disease, chronic diarrhea, necrotizing enterocolitis, and congenital enteropathy. Workup for celiac disease yielded negative results on two separate occasions, with T-transglutaminase IgA antibody levels <0.1 AU (a negative result being <5 AU).

Subsequently, the patient underwent genetic testing in the United States of America (USA), which revealed DGAT-1 enteropathy. Due to the child's malnourished state, management involved total parenteral nutrition via a central line for three days. His hydration status, fluid intake, and output were closely monitored through his electrolyte levels during the treatment process and there were no adverse or unanticipated events. The patient was well-adherent and tolerated the intervention well, as his signs of dehydration had diminished. The patient is currently on a protein-rich diet, consuming small portions multiple times a day at home. He was discharged from the hospital after three days, and as he traveled to the USA thereafter, the exact date of his follow-up is uncertain. However, we advised a follow-up after 14 days and requested follow-up investigations including CBC, LFTs, C-reactive protein (CRP), serum calcium, serum albumin, and 25-hydroxyvitamin D levels. His parents were informed about when to return to the hospital immediately and the red flags to watch for (severe diarrhea, failure to tolerate food or liquids, and a fever of 102°F or higher).

## Discussion

Diarrhea lasting more than two weeks is referred to as chronic diarrhea. Congenital diarrhea is the term used to describe a set of chronic diarrhea illnesses that can arise in the early stages of infancy. Numerous patients in this category suffer from monogenic illnesses that either directly impact the intestinal epithelium or indirectly impact the function of the intestinal epithelium [5]. While severe chronic diarrhea is the primary clinical manifestation for many of these illnesses, in others, diarrhea is just a symptom of a more complex multi-organ or systemic disease. In most cases, it is imperative to initiate adequate therapy right away in order to avoid dehydration and long-term problems that may even be fatal [6].

A decreased post-absorptive lipid profile is a result of acute pharmacological inhibition and systemic deletion of DGAT1, and this effect may be helpful in controlling postprandial incretin levels by altering the circulation and modifying lipid absorption, inhibiting DGAT1 in the enterocytes which increases GLP-1 levels. Furthermore, it has been shown that pharmacological inhibition of DGAT1 alters the type of lipids released into circulation and the pattern of lipid absorption in the small intestine with respect to time and location [7]. This could change the pattern of gut hormone secretion from entero-endocrine cells in the small intestine. Though the precise mode of action is still unknown, the significance of the DGAT1 enzymatic downstream effect on incretin release is becoming increasingly evident [8].

Although the exact mechanism of human DGAT1 deficiency-induced diarrhea is unknown, DGAT1 lipid substrate accumulation in the intestinal lumen or mucosa is probably a factor. Because fatty acid moieties behave as detergents or because they function as bioactive signaling lipids, excess diacylglycerols or fatty acids may become hazardous. Enteropathy with loss of protein could be caused by toxicity to enterocytes [9]. Diarrhea may result from bile acid malabsorption, and bile acid metabolism may be impacted by DGAT1 deficiency. Patients are difficult to detect in the early stages and frequently get misdiagnosed with infections, allergies, etc. since genetic abnormalities causing chronic diarrhea are clinically uncommon. Patients should get gene detection for a conclusive diagnosis if they show no discernible improvement following standard treatment [10].

For children with DGAT1 deficiency, there are currently no treatment medicines available to augment or restore intestinal DGAT function. The majority of diet plans are based on the verified experiences of a small number of patients [11]. Diets low in fat or fat-free are mostly used to reduce clinical symptoms; however, their effectiveness can vary depending on the mutation site and severity of protein synthesis dysfunction. Symptomatic therapies, including red blood cell transfusions, albumin and gamma-globulin supplements, and parenteral nourishment, are given during the acute phase [12].

## Conclusions

DGAT1 deficiency frequently exhibits severe symptoms at birth and during the neonatal period. Early diagnosis is essential since the disease might be fatal if treatment is not received. A strong suspicion of DGAT1 deficiency should be held for newborns with protein-losing enteropathy, growth retardation, and unexplained diarrhea and vomiting. An early diagnosis can be made with pathological intestinal investigations and early genetic identification.
